# Evaluation of a New Tandem Mass Spectrometry Method for Sickle Cell Disease Newborn Screening

**DOI:** 10.3390/ijns10040077

**Published:** 2024-11-26

**Authors:** Céline Renoux, Estelle Roland, Séverine Ruet, Sarah Zouaghi, Marie Michel, Philippe Joly, Cécile Feray, Fanny Zhao, Déborah Gavanier, Pascal Gaucherand, Fanny Roumieu, Giovanna Cannas, Salima Merazga, Philippe Connes, Gilles Renom, Jérôme Massardier, David Cheillan

**Affiliations:** 1 Laboratoire de Biologie Médicale Multi-Sites (LBMMS), Service de Biochimie et Biologie Moléculaire Grand Est, Hospices Civils de Lyon, 69500 Bron, France; celine.renoux@chu-lyon.fr (C.R.); severine.ruet@chu-lyon.fr (S.R.); sarah.zouaghi@chu-lyon.fr (S.Z.); marie.michel01@chu-lyon.fr (M.M.); philippe.joly@chu-lyon.fr (P.J.); cecile.feray@chu-lyon.fr (C.F.); fanny.zhao@chu-lyon.fr (F.Z.); 2 Centre Régional de Dépistage Néonatal Auvergne Rhône-Alpes (CRDN AuRA), 69003 Lyon, France; jerome.massardier@chu-lyon.fr; 3 Centre de Référence Constitutif des Maladies Constitutionnelles du Globule Rouge et de l’Erythropoïèse, 69003 Lyon, France; giovanna.cannas@chu-lyon.fr (G.C.); salima.merazga@chu-lyon.fr (S.M.); pconnes@yahoo.fr (P.C.); 4 Inter-University Laboratory of Human Movement Sciences, LIBM, Universite Claude Bernard Lyon 1, UR 7424, 69622 Villeurbanne, France; 5 Laboratoire de Dépistage Périnatal, Pôle de Biologie Pathologie Génétique, Centre Hospitalier Universitaire de Lille, 59000 Lille, France; estelle.roland@chu-lille.fr (E.R.); gilles.renom@chu-lille.fr (G.R.); 6 Service de Chirurgie Gynécologique et Cancérologique—Obstétrique, Centre Hospitalier Lyon Sud, Hospices Civils de Lyon, 69495 Pierre-Bénite, France; 7 Service d’Obstétrique, Hôpital Femme Mère Enfant, Hospices Civils de Lyon, 69500 Bron, France; pascal.gaucherand@chu-lyon.fr; 8 Service de Gynécologie-Obstétrique, Groupement Hospitalier Nord, Hospices Civils de Lyon, 69004 Lyon, France; fanny.roumieu@chu-lyon.fr; 9 Service de Médecine Interne, Hôpital Edouard Herriot, Hospices Civils de Lyon, 69003 Lyon, France; 10 Laboratoire Carmen—Inserm U1060, INRAE UMR1397, Université Claude Bernard Lyon 1, 69310 Pierre-Bénite, France

**Keywords:** newborn screening, sickle cell disease, tandem mass spectrometry

## Abstract

In France, sickle cell disease newborn screening (SCD NBS) has been targeted to at-risk regions since 1984, but generalization to the whole population will be implemented from November 2024. Although tandem mass spectrometry (MS/MS) is already used for the NBS of several inherited metabolic diseases, its application for SCD NBS has not been widely adopted worldwide. The aim of this study was to evaluate a dedicated MS/MS kit (Targeted MS/MS Hemo, ZenTech, LaCAR Company, Liege, Belgium) for SCD NBS and to compare the results obtained with those from an NBS reference center using matrix-assisted laser desorption/ionization time of flight (MALDI-TOF) and cation-exchange high-performance liquid chromatography (CE-HPLC, Variant NBS, Biorad Laboratories, Inc., Hercules, CA, USA) as confirmatory method. The MS/MS Hemo kit was used according to the manufacturer’s instructions and performed on a Waters Xevo TQ-D (Waters Corporation, USA). The software provided by the manufacturer was used for the calculation and analysis of peptide signal ratios. Among the 1333 samples, the results of 1324 samples were consistent with the HPLC and/or MALDI-TOF results (1263 FA, 50 FAS, 7 FAC, 1 FAO-Arab, and 3 FS). All the discordant results (one FAS on MS/MS vs. FA in CE-HPLC, one FA on MS/MS vs. FAS in CE-HPLC, seven FS on MS/MS vs. FAS in CE-HPLC) were corrected after modifying the peptide signal ratios thresholds, allowing the MS/MS Hemo kit to achieve near-100% sensitivity and specificity for SCD NBS. In conclusion, the MS/MS Hemo kit appears to be an effective method for SCD NBS, particularly for laboratories already equipped with MS/MS technology. However, these results should be confirmed in a larger cohort including a greater number of positive samples for SCD.

## 1. Introduction

Sickle cell disease (SCD) is a severe monogenic hemoglobinopathy characterized by the production of an abnormal hemoglobin (Hb), namely HbS [1]. When deoxygenated, HbS polymerizes and induces the sickling of red blood cells (RBCs). Sickle RBCs are more fragile and rigid than healthy RBCs, leading to chronic hemolytic anemia and frequent painful vaso-occlusive crisis [2,3,4]. SCD is the most common monogenic disease encountered around the world, with nearly 300,000 affected newborns annually. It is caused by either homozygosity for the β^S^ mutation (NM_000518.4(HBB):c.20A>T; p.(Glu7Val); SS) or by compound heterozygosity between the β^S^ mutation and a few types of β-globin abnormalities, HbC (SC), HbD-Punjab (SD-Punjab), HbO-Arab (SO-Arab), and HbE (SE), primarily, or a β-thalassemia mutation (Sβ-thal). The β^S^ mutation frequency is highly variable worldwide due to the natural selection imposed by malaria [5]. The highest frequency (>15%) is found in sub-Saharan Africa. The frequency is lower in the Middle East and India, with about 5% of carriers. In Europe, the mutation is rare, generally with a frequency of less than 1%, but the SCD prevalence in France is one of the highest in Europe, with nearly 25,000 SCD patients [6].

The clinical expression of SCD varies greatly from one patient to another, but the early initiation of preventive measures contributes to a strong reduction in morbidity and mortality during childhood [7]. In several European countries, newborn screening (NBS) for SCD has been, for a long time, a major element in a dedicated prevention program [8]. SCD NBS has been performed in French overseas departments since 1984, while it has been performed in mainland France since 2000, and only in at-risk newborns, according to the geographical origins of the parents. Currently, with the growing diversity of populations, targeted screening is no longer feasible, and SCD NBS will be generalized to the whole population in France from November 2024. In France, each SCD NBS reference center uses one method for a first-line screening of HbS. When positive, a second confirmatory method using another technology is required to assess a more precise screening. The SCD suspicion profiles FS, FSC, or FSX (where X designates another abnormal hemoglobin) are reported to the referring physician. For heterozygous carriers, family receive an information letter encouraging them to consider genetic counseling. Samples from premature or transfused newborns are not analyzed, and a new sample is requested.

Commonly used technologies include automated cation-exchange high-performance liquid chromatography (CE-HPLC), isoelectric focusing (IEF), and capillary electrophoresis (CE) [8,9]. In the last few years, two mass spectrometry methods dedicated to SCD have been developed: (i) a matrix-assisted laser desorption/ionization time of flight (MALDI-TOF) method focusing on the HbS molecule exclusively and (ii) a tandem mass spectrometry (MS/MS) method with the SpOtOn Clinical Diagnostics kit, as an example or with in-house method [10,11,12,13,14]. While MS/MS is already used for the NBS of several inherited metabolic diseases, this technology has never been used for SCD screening in France. The aim of this study was thus to compare the results of SCD NBS obtained with a dedicated MS/MS kit (Targeted MS/MS Hemo, ZenTech, LaCAR Company, Liege, Belgium) and the results obtained by a NBS reference center using MALDI-TOF as a first tier and CE-HPLC for confirmation of positive samples [13].

## 2. Materials and Methods

### 2.1. Sample Collection

One thousand four hundred and twenty-five newborns from the Hospices Civils de Lyon Maternity (Lyon, France) were included after information was given to the parents. Inclusions were limited to babies for whom SCD NBS was specifically conducted, targeting at-risk newborns based on the geographical origins of the parents. Seventy-seven patients were excluded because of insufficient quantity of dried blood on Guthrie cards. Residual dried blood spots (DBS) from standard Guthrie cards used for routine NBS program were collected from the 1348 remaining newborns included. Some clinical information (birth date, sex term, history of transfusion) was also noted. This study (DREPAMASSE; clinical trial number: NCT03985501) was conducted in agreement with the guidelines set by the Declaration of Helsinki, and was approved by the French Ethic committee (2019-A01346-51).

### 2.2. SCD Screening

At the time of the study, 13 diseases were screened for NBS French program (phenylketonuria, cystic fibrosis, congenital hypothyroidism, adrenal hyperplasia, sickle cell disease, and 7 inherited metabolic disorders) in a regional NBS laboratory. The Auvergne Rhône-Alpes region chose to delegate the SCD NBS to Lille University Hospital’s newborn screening laboratory (Lille, France) using MALDI-TOF as a first-tier method and CE-HPLC for confirmation of positive samples. During the study, samples were tested systematically by CE-HPLC in Lille, even if the first-tier method (MALDI-TOF) was negative. Residual blood spots from standard Guthrie cards used in Lyon NBS laboratory were punched (DBS Puncher, PerkinElmer, Waltham, MA, USA; diameter: 3.2 mm) into 96-well microplates. The MS/MS Hemo kit was used according to the manufacturer’s instructions, which included (i) a first step of extraction with water at room temperature (RT) for 15 min under stirring, (ii) a second step with a denaturation solution at RT for 10 min, and (iii) a third step of trypsin digestion at 37 °C for 2 h under stirring. Samples were diluted before injection into the mobile phase stream (ACN: H_2_O with 0.1% formic acid (50:50)) using a Waters Acquity I-Class and directly introduced into the source without any prior chromatographic separation. MS/MS analyses were performed in a triple quadrupole mass spectrometer (Xevo-TQ-D, Waters Corporation, Milford, MA, USA). Briefly, the extract was ionized by electrospray and hemoglobin peptides were separated by their mass-to-charge (m/z) ratios. The MS/MS mode used for this assay was the Multiple Reaction Monitoring (MRM) mode, in which two specific “parent ion/product ion” couples were analyzed, allowing the simultaneous detection of 12 peptides (Table 1): 7 corresponding to normal globins chains, α-peptide 1 (α^1^), α-peptide 2 (α^2^), β-peptide 1 (β^1^), β-peptide 2 (β^2^), β-peptide 3 (β^3^), δ-peptide1 (δ^1^), and γ-peptide 1 (γ^1^); and 5 corresponding to Hb variants, β-peptide S (β^S^), β-peptide C (β^C^), β-peptide E (β^E^), β-peptide O-Arab (β^O-Arab^), and β-peptide D-Punjab (β^D-Punjab^). Considering the time between each sample, the analysis time on the mass spectrophotomer is about 1.6 min.

Dedicated software, Targeted MSMS Hemo Software; Version 7.6 (H-LV-000), was provided by the manufacturer for the calculation of peptide signal ratios directly from the exported mass spectrometer data. Two algorithms, developed by the manufacturer, were defined and gave independent status for the alpha- and beta-globin chains. For this study, beta-globin interpretation only was considered, since the alpha-thalassemia status could not be confirmed without genetic consent. For the beta-globin chain, the following status could be assigned: (i) No Path. Detected (FA); (ii) HbS, HbC, HbDPunjab, HbE or HbO-Arab Carrier (FAS, FAC, FAD, FAE, or FAO, respectively); (iii) Sickle Cell Disease (FS) or HbSC (FSC); (iv) Prem-beta0, when suspected premature or β-thalassemia babies; and (v) Re-test, when the ratios obtained during the analysis cannot lead to a diagnosis by the software. This can be caused by invalid samples (issues during the preparation or analysis) or by inappropriate ratios. The interpretation provided by the algorithm is based on the complementary analysis of various peptide ratios, notably the β^S^/β^1^ ratio, to distinguish FA, FAS, and FS patients. Therefore, the means of the β^S^/β^1^ ratio were calculated for FA, FAS, and FS samples, with only concordant results from the three techniques considered.

### 2.3. Performance Analysis

A blood spot quality control (QC) included with the MS/MS Hemo kit and containing a mix of pathogenic peptides (β^S^, β^c^, β^D-Punjab^, β^E^, β^O-Arab^) was analyzed in each plate: 2 punches at the beginning (after the blank control, ie., a Guthrie card without dried blood), 2 punches in the middle, and 2 punches at the end. Between-series coefficients of variation (CV) were calculated using the values of the different peptides signal areas and peptides signal ratios from the same QC batch. Intra-series CV were also calculated after analysis of a FAS newborn sample 10 times in the same plate. Sensitivity and specificity for SCD NBS were calculated and compared to the results obtained from MS/MS, MALDI-TOF, and CE-HPLC. Since we did not have genetic confirmation, a result was considered certain if it was concordant with both CE-HPLC and MALDI-TOF methods. Negative results corresponded to FA patients, while positive results corresponded to FAS and FS patients.

## 3. Results

### 3.1. Performance Analysis

Four 96-sample-plates were analyzed with six quality controls (QC) in each plate (one plate/day) from the same QC batch (*n* = 24). When considering the peptide areas, the between-series CV obtained in the laboratory were above the expected values for every peptide, except for γ-peptide 1 (Table 2A). The results were thus interpreted using peptide area ratios determined for beta-globin interpretation, despite the absence of expected values from the manufacturer (Table 2B). For the β^1^/γ^1^, β^2^/γ^1^, and β^3^/γ^1^ ratios, the between-series CV ranged from 12.3% to 17.0%. For the Hb variant detection, the between-series CV were quite similar compared to the β/γ ratios, with values ranging from 10.0% to 21.9%.

For the intra-series CV of peptide areas for an A/S individual analyzed 10 times in a run, the values were once again above the expected values for every peptide area (Table 3). Regarding the β^S^/β^1^ ratio (which is the ratio used to detect HbS carriers), the intra-series CV was 7.5%, which is better than the corresponding between-series ratio.

These values were consistent with the national guidelines published by the NBS biology committee regarding MS/MS methods. This committee recommended that the intra-series and between-series CV should be less than 15% and 20%, respectively [15]. 

### 3.2. Samples Analysis: Sensitivity and Specificity

The comparison of the results obtained in the MS/MS versus MALDI-TOF and CE-HPLC methods are presented in Table 4. After the analysis of the 1348 remaining babies included in the study, the manufacturer’s algorithm was unable to reach a conclusive result and recommended a ‘re-test’ for 15 patients. However, there was not enough dried blood quantity to proceed a re-test. Thus, an interpretation was conducted for 1333 babies only.

The MS/MS results were compared to the CE-HPLC results. Among the 1260 FA patients in CE-HPLC, 1259 (99.9%) were FA in MS/MS, while 1 patient (<1%) was FAS. Among the 58 FAS patients identified by CE-HPLC, 50 (86.2%) were FAS, 7 (12.1%) were FS, and 1 (1.7%) was FA with the MS/MS Hemo kit. The values for all the FS, FAC, and FAO-Arab patients identified by CE-HPLC were correctly interpreted in MS/MS. One patient was identified as FAD-Punjab in CE-HPLC, and three were FAE, but no variant was detected in MS/MS.

The MS/MS results were also compared with the MALDI-TOF results. The MALDI-TOF method is currently configured to detect HbS only. Therefore, the eight FAX patients identified by MS/MS were not included in this comparison. Among the 1263 FA patients identified by MALDI-TOF, 1262 (99.9%) were FA and 1 (<1%) was FAS by MS/MS. Among the 59 FAS patients identified by MALDI-TOF, 50 (84.7%) were FAS, 7 (11.9%) were FS, and 2 (3.4%) were FA by MS/MS. All the FS patients determined by MALDI-TOF were correctly interpreted by MS/MS.

The β^S^/β^1^ ratio mean for the FA samples was 100 times lower (0.002 ± 0.01) than that for the FAS samples (1.05 ± 0.4), while the β^S^/β^1^ ratios for the three FS samples ranged between 6 and 79. The β^S^/β^1^ ratio for the 7 FAS patients misclassified as FS in MS/MS ranged between 0.8 and 1.1. These results are closer to the FAS ratio than to the FS ratio.

Finally, an interpretation was made considering only the detection of HbS, which is the essential parameter for SCD NBS. The results are presented in Table 5. The sensitivity and specificity of the MS/MS Hemo kit for HbS detection were 98.3% and 99.9%, respectively. The negative (NPV) and positive (PPV) predictive values were 99.9% and 98.3%, respectively.

## 4. Discussion

Because MS/MS is increasingly used for the NBS of inherited metabolic diseases, it would be convenient if the same equipment could also be used for SCD NBS. MS/MS is already used in other countries for SCD NBS, such as in England and Germany [11,14]. The between-series CVs fluctuated between 10.0% and 21.9%, which was higher than the expected values provided by the manufacturer. However, regardless of the method or technology used, it is common to fail to achieve the performance specifications of the manufacturer. In such cases, laboratories generally set their own CVs based on those obtained by peer groups. Regarding analytical performance, the intra-series β^S^/β^1^ ratio CV obtained for an AS patient (7.5%) was higher than the intra-assay CV obtained by another in-house MS/MS method (2.5%) [10] but fell within the same range as the one obtained with the SpotOne Clinical Diagnostics SCD NBS kit [11,15] and was even lower than the one reported by Hachani and al., who used the MALDI-TOF method [16]. The between-series CVs for the various peptide ratios obtained with the SpOtOn Clinical Diagnostics SCD NBS kit (London, UK) ranged from 8.9% to 21.2% [11] and fluctuated between 6.3% and 23.6% with another in-house MS/MS method [10]. These results were in agreement with those obtained by using the MS/MS Hemo kit. Despite the analytical performances not being those expected by the manufacturer, they were comparable to other MS/MS methods already in use and aligned with national guidelines (File S1). Like other SCD MS/MS protocols, the MS/MS Hemo kit analyses Hb peptides after trypsin digestion. Indeed, whole-protein analysis lacks specificity, since HbS is 30 Da lighter than the normal protein, and such a mass shift is observed for other abnormal Hb as well [10,11,15,17]. Similarly, other common Hb variants (HbC, HbE, HbD-Punjab, and Hb O-Arab) with mass differences of less than 1 Da could not be identified without trypsin digestion. In contrast to other methods, the MS/MS Hemo kit does not use an internal standard to check for the digestion step [11,15] or to perform semi-quantification [17]. Another in-house SCD MS/MS method did not use an internal standard [14]. Finally, good sensitivity and specificity could be obtained even in the absence of an internal standard.

NBS methods should be quick and easy to interpret. The use of an integrated algorithm greatly facilitated this step. Of the 1333 samples interpreted with the manufacturer algorithm, the results from 1324 samples were consistent with HPLC and/or MALDI-TOF results. The specificity of the MS/MS Hemo kit of near 100% is excellent and equivalent to the other methods used for SCD NBS [9,10,11,13,16]. Similarly, the negative predictive value of very close to 100% makes the MS/MS Hemo kit particularly applicable as a first-line SCD NBS. The sensitivity was comparable to the other methods used for SCD NBS [9,10,11,13,16]. Even if there was a low proportion of positive samples in our study, all the clinically relevant cases were correctly classified. Future studies should include a cohort with a higher number of positive samples. Among the nine discordant results, only one FAS sample appeared FA with the MS/MS Hemo kit and would have not been tested by another method in the normal course of the French SCD NBS program. This result was surprising, and the analysis was repeated for verification, with the patient’s identity checked to rule out a sampling error. No explanation has been found to date. It would be interesting to conduct genetic testing to possibly identify another molecular defect that has similar biochemical proprieties and leads to the false detection of a β^S^ peptide. The β^S^/β^1^ ratio for the seven FAS patients misclassified as FS are closer to the FAS ratio than to the FS ratio. The interpretation provided by the manufacturer’s algorithm is not based solely on the β^S^/β^1^ ratio, which could possibly explain this misclassification. Among these seven patients, four were premature HbS carriers (27 to 36 weeks) and were incorrectly classified as FS by the manufacturer’s algorithm due to a low HbA percentage. Moreover, numerous DBS samples were stored for over a year. At the beginning of the study, the HEMO MS/MS kit was still under development, and the instructions for use did not mention the pre-analytical storage duration for the samples. This point was later clarified by the manufacturer, and recommendations that storage should not exceed 1 month were included in the user manual. All the discordant results were corrected after the adaptation of the peptide ratio thresholds in collaboration with the manufacturer. These observations confirm the need to adapt the algorithm to the equipment used in each laboratory, as mentioned in the instructions for use of the Targeted MS/MS Hemo kit, and as recommended by Moat et al. with the SpOtOn Diagnostics Kit [11]. Thus, while algorithms are helpful tools for interpretation, the vigilance of a biologist remains essential for the correct interpretation of results.

While the MS/MS Hemo kit could also detect HbC, HbE, HbD-Punjab, and HbO-Arab, the three samples identified with an HbE variant and the sample identified with an HbD-Punjab in CE-HPLC were not detected with the MS/MS Hemo kit. Since three different phenotypic techniques are needed to identify an Hb variant, these variants observed in CE-HPLC could correspond to other variants migrating in the same zone of HbE or HbD-Punjab. A confirmatory phenotypic or genotypic analysis is necessary to confirm the presence of HbE or HbD-Punjab in these samples, but also to confirm the true phenotype in case of discordant results.

In conclusion, the MS/MS Hemo kit provides reliable detection of abnormal Hb variants, with an analytical performance comparable to those of other MS/MS methods. It is an efficient approach for SCD NBS, optimizing the use of equipment and expertise already available in most NBS laboratories worldwide. However, it is important that each laboratory adapts the threshold ratios in the manufacturer’s algorithm, taking into account results confirmed by molecular biology, as far as possible, to minimize interpretation errors. Furthermore, further studies with a large number of positive samples could improve the sensitivity of the method. Finally, the presence of all variants should be verified using a secondary method, as recommended in the majority of SCD NBS programs.

## Figures and Tables

**Table 1:** Parent and product ions for each screened hemoglobin peptides.

Targeted Compound	Parent Ion (*m*/*z*)	Product Ion (*m*/*z*)
α-peptide 1	536.5	446.4
		680.3
α-peptide 2	627	233.4
		261
β-peptide 1	477	237
		502.3
β-peptide 2	658	313
		758.6
β-peptide 3	690	378
		501
β-peptide S	462	237.2
		472.1
β-peptide C	694.5	237.1
		244.2
β-peptide E	459	214.2
		360.2
β-peptide OArab	625.6	249
		501.1
β-peptide DPunjab	689.6	377.1
		276.1
γ-peptide 1	550	251.3
		634.2

**Table 2:** Between-series CV from quality controls considering either peptide areas (**A**) or peptide areas ratios (**B**).

**(A) Peptide Areas**	**β^1^**	**α^1^**	**γ^1^**	**α^2^**	**β^2^**	**β^3^**	**β^S^**	**β^E^**	**β^C^**	**β^D^**	**β^O^**	**δ^1^**
Laboratory CV (%)	21	27	18	30	22	23	23	34	20	19	24	42
Manufacturer CV (%)	9.9	10.4	18.4	17.7	13.8	13.4	8.6	NA	NA	NA	NA	NA
**(B) Peptide Areas Ratios**		**β** **^1^** **/** **γ** **^1^**	**β** **^2^** **/** **γ** **^1^**		**β** **^3^** **/** **γ** **^1^**	**β^s^** **/** **β^1^**		**β^c^** **/** **β^1^**	**β^E^** **/** **β^1^**	**β^D^** **/** **β^1^**		**β^O^** **/** **β^1^**
Laboratory CV (%)		13.2	12.3		17.0	11.8		15.0	21.9	10.0		11.9
Manufacturer CV (%)		NA	NA		NA	NA		NA	NA	NA		NA

**Table 3:** Intra-series CV for peptide areas after AS patient analyses (*n* = 10).

	β^1^	α^1^	γ^1^	α^2^	β^2^	β^3^	β^S^	β^s^/β^1^
Laboratory CV (%)	5.6	10.0	60.4	18.4	10.4	8.4	6.4	7.5
Manufacturer CV (%)	4.8	6.4	7.1	6.9	5.7	4.7	3.4	NA

**Table 4:** Comparison of the results obtained with MS/MS and MALDI-TOF or CE-HPLC methods.

	MS/MS Concordance with								Discrepancies		
	CE-HPLC and MALDI-TOF			CE-HPLC Only			MALDI-TOF Only				
MS-MS Hemo kit (ZenTech, Liege, Belgium)	FA	FAS	FS	FA	FAO-Arab	FAC	FA	FA	FAS	FA	FS
MALDI-TOF	FA	FAS	FS	FAS	FA	FA	FA	FA	FA	FAS	FAS
CE-HPLC (Variant NBS, Biorad, Hercules, CA, USA)	FA	FAS	FS	FA	FAO-Arab	FAC	FAE	FAD	FA	FAS	FAS
	1258	50	3	1	1	7	3	1	1	1	7

FA: no hemoglobinopathy; FAS: HbS carrier, FS: suspicion of sickle cell anemia; FAO-Arab: Hb O-Arab carrier; FAC: HbC carrier; FAE: HbE carrier; FAD: Hb D-Punjab carrier.

**Table 5:** Comparison of the results obtained in MS/MS considering HbS detection only.

Negative Samples *		Positive Samples *	
True	False	True	False
1258	1	60	1
1259		61	

* Negative samples correspond to FA patients and positive samples correspond to FAS and FS patients. Since we did not have genetic confirmation, a result was considered certain if it was obtained by both CE-HPLC and MALDI-TOF methods.

## Data Availability

The data that support the findings of this study are available from the corresponding author upon reasonable request.

## Supplementary Materials

The following supporting information can be downloaded at: https://www.mdpi.com/article/10.3390/ijns10040077/s1. File S1: Recommendations for MSMS method for newborn screening.
