# Supplementary material for: Evaluation of a New Tandem Mass Spectrometry Method for Sickle Cell Disease Newborn Screening
Source: Int J Neonatal Screen. 2024 Nov 26;10(4):77. doi: 10.3390/ijns10040077 (PMC11676960; doi:10.3390/ijns10040077)
Supplement: Supplementary file 1 [file IJNS-10-00077-s001.zip › IJNS-3251155-supplementary.pdf]

Recommendations for MSMS method for newborn screening

Extract and translation of the French document

Working group members : Paul BREGEAUT, Christelle CORNE, David GUENET, Cindy LAURO, Samir MESLI, Thao NGUYEN KHOA, Gilles RENOM, Sarah ROMAIN

III-1/ Fidelity verification

Intra-series

The acceptable coefficient of variation (CV) must be less than 15%, as indicated by previous method validation results.

Inter-series :

The acceptable CV must be less than 20%, based on prior method verification.

These results align with international recommendations: a 15% threshold is specified for chromatographic methods (for both repeatability and intermediate precision), and a 20% threshold is acceptable when the expected value is near the limit of quantification.

For further details, refer to the *Bioanalytical Method Validation – Guidance for Industry – May 2018*.
